# Editorial for Special Issue: iPS Cells (iPSCs) for Modelling and Treatment of Human Diseases

**DOI:** 10.3390/cells11152270

**Published:** 2022-07-22

**Authors:** Nina Graffmann, James Adjaye

**Affiliations:** Institute for Stem Cell Research and Regenerative Medicine, Medical Faculty, Heinrich-Heine University, 40225 Dusseldorf, Germany

Human induced pluripotent stem cells (iPSCs) have evolved as a powerful tool to model diseases and study treatment possibilities [1] iPSCs have many advantages over classical disease models, such as animals, cell lines or patient-derived primary cells. They can be generated from any cell of the adult body with limited inconvenience for the patient, especially when urine cells are used as a starting material. Thus, it is easy to obtain cells from any desired genetic background; additionally, rare diseases can be studied using these cells. Using genome-editing techniques, isogenic controls and reporter cell lines can be obtained. These enable the investigation of the genetic basis for certain diseases, and reporter cell lines ensure that the desired cell type can be studied in isolation, as well as in combination with other relevant cell types for the organ. Being of human origin, they have the potential to outperform animal studies with respect to toxicological predictions, as drug metabolism varies substantially between species. However, until now, the lack of maturity of in vitro differentiated cells has limited their use in this field, and more research is needed to close this gap.

In this Special Issue, we have collected articles that emphasise the wide application possibilities of iPSCs. The publications cover diseases of brain [2,3,4,5], kidney [6], blood [7,8], cartilage [9] and primordial germ cells [10] in 2D and 3D models.

Four publications focus on brain diseases. With their models, Halliwell et al. and Nouri et al. provide valuable ground-work for studying brain diseases. Nouri et al. generated a NESTIN–mScarlet red-fluorescent-reporter human iPSC line that reliably marks neural stem/progenitor cells (NSCs/NPCs) during in vitro development [5]. Using this cell line, it is possible to identify cells of neuronal lineage in the mixed cell cultures that appear during in vitro brain development and separate them via flow-cytometric cell sorting for further analyses. Halliwell et al. systematically studied the electrophysiological profile of iCell neurons via multi-electrode analysis (MEA) and patch-clamp technology [3]. They demonstrated that after about one week of culture, a dominant inhibitory tone from GABAergic neurons appeared. Various drugs could influence the neuronal signaling, which emphasises the model’s usefulness in drug development studies.

Martins et al. employed 3D brain organoids to model the rare neurodevelopmental disease Nijmegen Breakage syndrome (NBS) [4]. This disease is caused by mutations in the DNA double-strand repair gene nibrin. They showed that patient-derived organoids are significantly smaller and have disrupted cytoarchitecture. In addition, they differentiated in a premature manner compared to wild-type organoids. Overall, these organoids could reproduce the microcephaly phenotype, which is a hallmark of NBS. Furthermore, DNA damage-response and cell-cycle genes were differentially regulated compared to controls, underlining the role of nibrin for DNA repair.

While these three manuscripts describe fundamental research for disease modelling, the study by Armijo et al. provides insights into the possible application of iPSC derived neural precursor cells as a therapeutic agent. They transplanted mouse iPSC-derived neural precursor cells into the brains of mice suffering from pathological abnormalities typical of Alzheimer’s disease [2]. After transplantation, their memory and synaptic plasticity improved, and the typical pathophysiological signs of Alzheimer’s disease such as amyloid-beta plaques and tangle deposits were reduced. Although this study did not feature human cells, it provides hope for future treatment options.

Two publications in this Special Issue used iPSCs to model blood-associated diseases. Qanash et al. tested several drugs as potential treatments for Diamond–Blackfan anaemia (DBA) in an iPSC-based differentiation model [8]. Globin synthesis is delayed in DBA due to ribosomal dysfunctions. Thus, patients suffer from excess toxic free haem in erythroid progenitors, early differentiation arrest and pure red-cell aplasia. By comparing patient-derived cells and isogenic controls generated by CRISPR/cas9, the authors demonstrated that molecules with intracellular iron-chelating properties that restrict the labile iron pool within the cells, such as eltrombopag, could rescue erythropoiesis in the patient-derived cells. Chelators that do not affect the labile iron pool did not show any beneficial effects.

The review by Pratumkaew et al. provides deeper insights into the potential of iPSCs in modelling acquired and hereditary blood diseases, as well as for their therapeutic applications [7].

Rim et al. provide a proof-of-concept iPSC-based model for early-onset osteoarthritis. To this end, they generated iPSCs from a patient and a healthy sibling and compared these cells during chondrogenic differentiation [9]. They demonstrated that patient-derived chondrogenic pellets are bigger and contain more vacuoles than those of a healthy origin. In addition, they observed differences in the expression of disease-relevant genes and the secretion of inflammatory cytokines. With this novel iPSC-based model, the early stages of osteoarthritis, for which no primary tissue is usually available, can be studied effectively.

Another difficult-to-study disease is Turner syndrome, which is caused by X-chromosome monosomy in females. The molecular basis for the associated premature primary gonadal failure in these patients is especially poorly understood. De Souza et al. generated iPSCs from Turner Syndrome patients, which are suitable for modelling early germline development and resemble primary primordial germ cells regarding transcripts, protein marks and the epigenetic profile [10].

The last paper in this Special Issue investigated the usefulness of iPSC-derived kidney organoids to model kidney injuries. Nguyen et al. used urine stem-cell-derived iPSCs to generate kidney organoids with distinct glomerular and tubular regions [6]. They demonstrated that upon treatment with the nephrotoxin puromycin aminonucleoside (PAN), both regions show morphological damage. Transcriptome analyses confirmed that PAN treatment induces kidney damage via a network of inflammation, cytoskeletal re-arrangement, DNA damage, apoptosis and cell death.

Taken together, these publications use iPSCs to study a wide field of diseases, encompassing several organ systems. They prove the usefulness of iPSCs in modelling diseases without the need for primary patient-derived cells from the affected tissue. Instead, it is sufficient to use easily accessible primary cells such as urine cells as a starting material for iPSC generation. All the models reproduced the functional hallmarks of the cells, as well as disease-associated features. Damage known to be caused by specific drugs and toxins could be reproduced, which underlines the usefulness of these systems for further studies in the field of drug development and toxicity.
